# Supplementary material for: A retrospective approach for evaluating ecological niche modeling transferability over time: the case of Mexican endemic rodents
Source: PeerJ. 2024 Nov 29;12:e18414. doi: 10.7717/peerj.18414 (PMC11610470; doi:10.7717/peerj.18414)
Supplement: Supplementary file 1 [file peerj-12-18414-s001.odt]

Supplementary Material

Table S1. List of the Mexican endemic rodents with conservation status according to Mexican law (NOM-059-SEMARNAT-2010). NOM-059 species classification: Pr (Subject to special protection), A (Endangered), E (Probably extinct in the wild), P (Endangered species). Distribution: Co (Continental), In (Insular).

| Family | Species | Conservation Status | Distribution | Modeled |
| --- | --- | --- | --- | --- |
| Agoutidae | Dasyprocta mexicana (de Saussure, 1861) |  | Co | x |
| Cricetidae | Habromys chinanteco (Robertson y Musser, 1976) |  | Co | - |
| Habromys delicatulus (Carleton, Sánchez, y Urbano Vidales, 2002) |  | Co | - |
| Habromys ixtlani (Goodwin, 1964) |  | Co | - |
| Habromys lepturus (Merriam, 1898) |  | Co | - |
| Habromys schmidly (Romo-Vázquez, León Paniagua, and Sánchez, 2005) |  | Co | - |
| Habromys simulatus (Osgood, 1904) | Pr | Co | - |
| Hodomys alleni (Meriam, 1892) |  | Co | - |
| Megadontomys cryophilus (Musser, 1964) | A | Co | x |
| Megadontomys nelsoni (Meriam, 1898) | A | Co | - |
| Megadontomys thomasi (Merriam, 1898) | Pr | Co | - |
| Microtus oaxacensis (Goodwin, 1966) | A | Co | x |
| Microtus quasiater (Coues, 1874) | Pr | Co | x |
| Microtus umbrosus (Merriam, 1898) | Pr | Co | - |
| Nelsonia goldmani (Merriam, 1897) | Pr | Co | - |
| Nelsonia neotomodon (Merriam, 1897) | Pr | Co | - |
| Neotoma angustapalata (R.H.Baker, 1951) |  | Co | - |
| Neotoma bryanti (Merriam, 1887) | A | Co-In | - |
| Neotoma goldmani (Merriam, 1903) |  | Co | x |
| Neotoma insularis (Townsend, 1912) | A | In | - |
| Neotoma nelsoni (Goldman, 1905) |  | Co | - |
| Neotoma palatina (Goldman, 1905) |  | Co | - |
| Neotoma phenax (Merriam, 1903) | Pr | Co | - |
| Neotomodon alstoni (Merriam, 1898) |  | Co | x |
| Oryzomys albiventer (Merriam, 1901) |  | Co | x |
| Oryzomys chapmani (Thomas, 1898) | Pr | Co | x |
| Oryzomys guerrerensis (Goldman, 1915) |  | Co | x |
| Oryzomys melanotis (Thomas, 1893) |  | Co | x |
| Oryzomys nelsoni (Merriam, 1898) | E | In | - |
| Oryzomys peninsulae (Thomas, 1897) | E | Co | - |
| Osgoodomys banderanus (J.A. Allen, 1897) |  | Co | x |
| Peromyscus bullatus (Osgood, 1904) | Pr | Co | - |
| Peromyscus caniceps (Burt, 1932) | Pr | In | - |
| Peromyscus carletoni (Bradley, Ordoñez-Garza, Sotero Caio, Huynh et al. 2014) |  | Co | - |
| Peromyscus dickeyi (Burt, 1932) | Pr | In | - |
| Peromyscus difficilis (Allen, 1891) |  | Co | x |
| Peromyscus eva (Thomas, 1898) | A | Co | x |
| Peromyscus furvus (J.A. Allen y Chapman, 1897) |  | Co | x |
| Peromyscus guardia (Townsend, 1912) | P | In | - |
| Peromyscus hooperi (Lee y Schmidly, 1977) |  | Co | - |
| Peromyscus hylocetes (Merriam, 1898) |  | Co | x |
| Peromyscus interparietalis (Burt, 1932) | A | In | - |
| Peromyscus latirostris (Dalquest, 1950) |  | Co | - |
| Peromyscus levipes (Merriam, 1898) |  | Co | x |
| Peromyscus madrensis (Merriam, 1898) | A | In | - |
| Peromyscus megalops (Merriam, 1898) |  | Co | x |
| Peromyscus mekisturus (Merriam, 1898) | A | Co | - |
| Peromyscus melanocarpus (Osgood, 1904) |  | Co | x |
| Peromyscus melanophrys (Coues, 1874) |  | Co | x |
| Peromyscus melanurus (Osgood, 1909) |  | Co | - |
| Peromyscus ochraventer (R.H.Baker, 1951) |  | Co | - |
| Peromyscus pembertoni (Burt, 1932) | E | In | - |
| Peromyscus perfulvus (Osgood, 1945) |  | Co | x |
| Peromyscus polius (Osgood, 1904) |  | Co | - |
| Peromyscus pseudocrinitus (Burt, 1932) | A | In | - |
| Peromyscus sagax (Elliot, 1903) |  | Co | - |
| Peromyscus schmidlyi (Bradley, Carroll, Haynie, Muníz-Martínez et al., 2004) |  | Co | - |
| Peromyscus sejugis (Burt, 1932) | A | In | - |
| Peromyscus simulus (Osgood, 1904) |  | Co | - |
| Peromyscus slevini (Mailliard, 1924) | A | In | - |
| Peromyscus spicilegus (J.A.Allen, 1897) |  | Co | - |
| Peromyscus stephani (Townsend, 1912) | A | In | - |
| Peromyscus winkelmanni (Carleton, 1977) | Pr | Co | - |
| Peromyscus zarhynchus (Merriam, 1898) | Pr | Co | x |
| Reithrodontomys bakeri (Bradley, Mendez-Harclerone, Hamilton y Ceballos 2004) |  | Co | - |
| Reithrodontomys burti (Benson, 1939) |  | Co | - |
| Reithrodontomys chrysopsis (Merriam, 1900) |  | Co | x |
| Reithrodontomys hirsutus (Meriam, 1901) |  | Co | - |
| Reithrodontomys spectabilis (J.K.Jones y Lawlor, 1956) | A | In | - |
| Reithrodontomys zacatecae (Merriam, 1901) |  | Co | - |
| Rheomys mexicanus (Goodwin, 1959) | Pr | Co | - |
| Sigmodon alleni (Bailey, 1902) |  | Co | x |
| Sigmodon leucotis (Bailey, 1902) |  | Co | x |
| Sigmodon mascotensis (J.A.Allen, 1897) |  | Co | x |
| Sigmodon planifrons (Nelson y Goldman, 1933) |  | Co | - |
| Tylomys bullaris (Merriam, 1901) | A | Co | - |
| Tylomys tumbalensis (Merriam, 1901) | Pr | Co | - |
| Xenomys nelsoni (Merriam, 1892) | A | Co | - |
| Geomyidae | Cratogeomys fulvescens (Merriam, 1895) |  | Co | - |
| Cratogeomys fumosus (Merriam, 1892) | A | Co | x |
| Cratogeomys goldmani (Merriam, 1895) |  | Co | x |
| Cratogeomys merriami (Thomas, 1893) |  | Co | x |
| Cratogeomys perotensis (Merriam, 1895) |  | Co | - |
| Cratogeomys planiceps (Merriam, 1895) |  | Co | - |
| Geomys tropicalis (Goldman, 1915) | A | Co | - |
| Orthogeomys cuniculus (Elliot, 1905) | A | Co | - |
| Orthogeomys lanius (Elliot, 1905) | A | Co | - |
| Pappogeomys bulleri (Thomas, 1892) |  | Co | x |
| Thomomys atrovarius (J.A.Allen, 1898) |  | Co | x |
| Thomomys nayarensis (Mathis, Hafner, Hafner y Demastes, 2013) |  | Co | - |
| Thomomys sheldoni (Bailey, 1915) |  | Co | - |
| Zygogeomys trichopus (Merriam, 1895) | P | Co | - |
| Heteromyidae | Chaetodipus ammophilus (Osgood, 1907) | A | Co | - |
| Chaetodipus arenarius (Merriam, 1894) | A | Co | x |
| Chaetodipus artus (Osgood, 1900) |  | Co | x |
| Chaetodipus goldmani (Osgood, 1900) |  | Co | x |
| Chaetodipus lineatus (Dalquest, 1951) |  | Co | - |
| Chaetodipus pernix (J.A.Allen, 1898) |  | Co | x |
| Chaetodipus siccus (Osgood, 1907) | A | Co-In | - |
| Dipodomys gravipes (Huey, 1951) | E | Co | - |
| Dipodomys nelsoni (Merriam, 1907) |  | Co | x |
| Dipodomys ornatus (Merriam, 1894) |  | Co | x |
| Dipodomys phillipsii (Gray, 1841) | Pr | Co | x |
| Heteromys spectabilis (Genoways, 1971) | Pr | Co | - |
| Heteromys temporalis (Goldman, 1911) |  | Co | - |
| Sciuridae | Callospermophilus madrensis (Merriam, 1901) | Pr | Co | x |
| Cynomys mexicanus (Merriam, 1892) | P | Co | x |
| Neotamias bulleri (J.A. Allen, 1889) |  | Co | - |
| Neotamias durangae (J.A. Allen, 1903) |  | Co | x |
| Neotamias solivagus (A.H. Howell, 1922) |  | Co | - |
| Notocitellus adocetus (Merriam, 1903) |  | Co | x |
| Notocitellus annulatus (Audubon y Bachman, 1842) |  | Co | x |
| Otospermophilus atricapillus (Bryant,1889) |  | Co | - |
| Sciurus alleni (Nelson, 1898) |  | Co | x |
| Sciurus colliaei (Richardson, 1839) |  | Co | x |
| Sciurus oculatus (Peters, 1863) | Pr | Co | - |
| Tamiasciurus mearnsi (Townsend, 1897) | A | Co | - |

Table S2. List of sources consulted to obtain ocurrence records of the Mexican endemic rodents.

Aguilar-Lopez, M., 2007. Uso diferencial del espacio entre sexos y dinámica poblacional de Neotomodon alstoni (Rodentia: Muridae) en el cerro del Ajusco, México. Tesis Profesional. Pachuca, Hidalgo, México.

Álvarez-Castañeda, S. T. 2007. Creación de la base de datos de los Parques Nacionales Sierra de San Pedro Mártir y Constitución de 1857, y de la Reserva de la Biosfera Islas del Golfo de California para el grupo de los mamíferos. Centro de Investigaciones Biológicas del Noroeste S.C., Bases de datos SNIB-CONABIO proyecto No. BK011 México D. F.

Álvarez-Castañeda, S. T. 2008. Computarización de la Colección de mamíferos del Centro de Investigaciones Biológicas del Noroeste SC. Centro de Investigaciones Biológicas del Noroeste. Bases de datos SNIB-CONABIO proyecto No.CC001. México D. F.

Álvarez-Solorzano, T., J. C. López-Vidal. 1998. Biodiversidad de los mamíferos en el Estado de Michoacán. Instituto Politécnico Nacional. Escuela Nacional de Ciencias Biológicas. Base de datos SNIB-CONABIO proyecto No. P020. México D. F.

Angelo State Natural History Collections (ASNHC): Angelo State Natural History Collections (ASNHC) - Mammalogy Collection.

Arctos, Division of Mammals, Museum of Southwestern Biology, Albuquerque, NM (accessed through GBIF data portal, https://www.gbif.org).

Arctos, MVZ Mammal Catalog (accessed through GBIF data portal, https://www.gbif.org).

Arroyo-Cabrales, J., L. León-Paniagua. 2010. Catálogo de los mamí­feros de México en resguardo de The Natural History Museum (London), Inglaterra. Instituto Nacional de Antropología e Historia. Universidad Nacional Autónoma de México. Bases de datos SNIB-CONABIO, proyecto No. DE020. México, D. F.

Briones-Salas, M. A. 2006. Incorporación de nuevos registros de mamíferos de la región Sierra Norte de Oaxaca. Instituto Politécnico Nacional. Centro Interdisciplinario de Investigación para el Desarrollo Integral Regional-Oaxaca. Bases de datos SNIB-CONABIO proyectos No. BA009 y R104. México, D.F.

California Academy of Sciences, CAS Mammalogy (MAM) (accessed through GBIF data portal, https://www.gbif.org).

Ceballos, G. 2002. Actualización de la base de datos del Atlas Mastozoológico de México. Universidad Nacional Autónoma de México. Instituto de Ecología. Bases de datos SNIB-CONABIO proyectos No. T009 y A003. México, D.F.

Cervantes-Reza, F. A. 2000. Sistemática y biogeografía del género Reithrodontomys (Rodentia: Muridae). Universidad Nacional Autónoma de México. Instituto de Biología. Bases de datos SNIB-CONABIO proyecto No. L313. México, D.F.

Division of Mammals, Museum of Southwestern Biology (MSB) (accessed through SNIB CONABIO).

Escobar-Ocampo, M. C., Morales-Pérez, J. E., Hernández-García, E., Guzmán-Hernández, J., Riechers Pérez, A., Espinoza-Medinilla, E. E. 2006. Sistematización de las colecciones científicas del Instituto de Historia Natural y ecología, (IHNE) Chiapas. Instituto de Historia Natural y Ecología. Bases de datos SNIB-CONABIO. IHNE_Vertebrados proyectos No. V050, L018 y P060. México, D.F.

Field Museum, Field Museum of Natural History (Zoology) Mammal Collection (accessed through GBIF data portal, https://www.gbif.org).

García-Mendoza, D. F. 2015. Actualización de la base de datos de la Colección Regional Durango (Mammalia). Centro Interdisciplinario de Investigación para el Desarrollo Integral Regional Durango. Instituto Politécnico Nacional. Bases de datos de ejemplares SNIB-CONABIO proyecto No. HA002, DC015. México, D.F.

García-Mendoza, D. F. 2015. Actualización de la base de datos de la Colección Regional Durango (Mammalia). Centro Interdisciplinario de Investigación para el Desarrollo Integral Regional Durango. Instituto Politécnico Nacional. Bases de datos de tejidos SNIB-CONABIO proyecto No. HA002. México, D.F.

Global Biodiversity Information Facility [GBIF] (2018) GBIF occurrence download Mammalia. https://www.gbif.org/occurrence/download/0000381-180508205500799

González-Cózatl, F. X. 2012. Computarización de la Colección de Mamíferos del Centro de Educación Ambiental e Investigación Sierra de Huautla (CEAMISH) de la Universidad Autónoma del Estado de Morelos (UAEM). Universidad Autónoma del Estado de Morelos. Centro de Investigación en Biodiversidad y Conservación. Bases de datos SNIB-CONABIO proyecto No. HC022. México D. F.

Gual, D. M., Rendón, C. A., Alamilla, F. L.., Cifuentes, R. P., Lozano, R. A. T. 2013. Bosque Mesófilo de Montaña de México. Comisión Nacional para el Conocimiento y Uso de la Biodiversidad. Base de datos SNIB-CONABIO proyecto Sistema de Información del Bosque Mesófilo de Montaña en México (Sl-BMM). México, D. F.

Hortelano-Moncada, Y., Cervantes F.A., Vargas-Cuenca, J. 2016. Código de barras de ejemplares de la Colección Nacional de Mamíferos del Instituto de Biología, UNAM. Universidad Nacional Autónoma de México. Instituto de Biología. Base de datos SNIB-CONABIO proyecto No. HB029. México, D.F.

Kariwa, H., Yoshida, H., Sánchez-Hernández, C., Romero-Almaraz, M.D.L., Almazán-Catalán, J.A., Ramos, C., Miyashita, D., Seto, T., Takano, A., Totani, M., Murata, R., Saasa, N., Ishizuka, M., Sanada, T., Yoshii, K., Yoshimatsu, K., Arikawa, J., Takashima, I., 2012. Genetic diversity of hantaviruses in Mexico: Identification of three novel hantaviruses from Neotominae rodents. Virus Res. 163, 486–494. doi:10.1016/j.virusres.2011.11.013

León, P. L.., Ávila, V.Z.A..2012. Actualización de la base de datos de la Colección de mamíferos del Museo de Zoología 'Alfonso L. Herrera'. Universidad Nacional Autónoma de México. Facultad de Ciencias. Bases de datos SNIB-CONABIO, proyectos EC010, M099_Mamíferos, J123_Mamíferos, H160, B002_Mamíferos. México D. F.

López-González, C. 2012. Mamíferos silvestres de la cuenca del río Mezquital-San Pedro, Durango-Nayarit. Instituto Politécnico Nacional. Centro Interdisciplinario de Investigación para el Desarrollo Integral Regional-Durango. Bases de datos SNIB-CONABIO, proyecto No. GT015. México D. F.

López-González, C., Espinosa-Pérez, H. S. 2004. Peces y Mamíferos de la región de Norogachi, Alta Sierra Tarahumara, Chihuahua. Instituto Politécnico Nacional. Centro Interdisciplinario de Investigación para el Desarrollo Integral Regional-Durango. Bases de datos SNIB-CONABIO. Proyecto No. X011. México, D.F.

López Vidal, J. C. 2006. Computarización de las colecciones de vertebrados terrestres de la Escuela Nacional de Ciencias Biológicas, IPN Fase 1: Estado de México, Hidalgo, San Luis Potosí y Tlaxcala. Instituto Politécnico Nacional. Escuela Nacional de Ciencias Biológicas. Bases de datos SNIB-CONABIO. Proyecto No. BC004. México, D.F.

López Wilchis, R. 1998. Base de datos de mamíferos de México depositados en colecciones de Estados Unidos y Canadá. Universidad Autónoma Metropolitana. Unidad Iztapalapa. Bases de datos SNIB-CONABIO. Proyecto No. P130. México, D.F.

Lopéz-Vidal, J. C., Elizalde-Arellano, C., Campos-Rodríguez, J. I., Matás-Ferrer, N., González, N., Murillo-Jiménez, S. 2008. Computarización de las colecciones de vertebrados terrestres de la Escuela Nacional de Ciencias Biológicas, IPN - Fases 2 y 3. Instituto Politécnico Nacional. Escuela Nacional de Ciencias Biológicas. Bases de datos SNIB-CONABIO. Fase 3. Proyecto No. CC002. México, D.F.

Lopéz-Vidal, J. C., Elizalde-Arellano, C., Campos-Rodríguez, J. I., Matás-Ferrer, N., González, N., Murillo-Jiménez, S. 2008. Computarización de las colecciones de vertebrados terrestres de la Escuela Nacional de Ciencias Biológicas, IPN - Fases 2 y 3. Instituto Politécnico Nacional. Escuela Nacional de Ciencias Biológicas. Bases de datos SNIB-CONABIO. Fase 2. Proyecto No. CC002. México, D.F.

Louisiana State University Museum of Natural Science, Mammal specimens (accessed through GBIF data portal, https://www.gbif.org).

Martínez Vázquez, J. 2001. Mastofauna de la vertiente occidental (oeste) del Parque Nacional Pico de Orizaba, Puebla (Fase 1). Benemérita Universidad Autónoma de Puebla. Escuela de Biología. Bases de datos SNIB-CONABIO proyecto No. R044. México, D.F.

Michigan State University Museum, Vertebrate specimens (accessed through GBIF data portal, https://www.gbif.org).

Moore Laboratory of Zoology (MLZ) (accessed through SNIB CONABIO).

MSU Mammalogy, Ornithology and Vertebrate Paleontology Collections (accessed through SNIB CONABIO).

Muñiz Martínez, R. 2001. Vertebrados terrestres de San Juan de Camarones, Durango. Instituto Politícnico Nacional. Centro Interdisciplinario de Investigación para el Desarrollo Integral Regional-Durango. Bases de datos SNIB-CONABIO proyecto No. R008. México, D.F.

Muñoz-Alonso, L. A., March-Mifsut, I. J. 2003. Actualización y enriquecimiento de las bases de datos del proyecto de evaluación y análisis geográfico de la diversidad faunística de Chiapas. El Colegio de la Frontera Sur. Bases de datos SNIB-CONABIO proyectos No. U014 y P132. México, D.F.

Museum of Comparative Zoology, Harvard University, Museum of Comparative Zoology, Harvard University (accessed through GBIF data portal, https://www.gbif.org).

Museum of Texas Tech University (TTU), Mammal specimens (accessed through GBIF data portal, https://www.gbif.org).

Museum of Vertebrate Zoology (MVZ), University of California, Berkeley (accessed through SNIB CONABIO).

National Museum of Natural History, Smithsonian Institution, NMNH Vertebrate Zoology Mammals Collections (accessed through GBIF data portal, https://www.gbif.org).

National Museum of Natural History, Smithsonian Institution: NMNH occurrence DwC-A

New Mexico Museum of Natural History and Science, NMMNH Mammal (accessed through GBIF data portal, https://www.gbif.org).

Ramírez-Pulido, J. 1999. Biodiversidad mastozoológica del Eje Volcánico Transversal. Universidad Autónoma Metropolitana-Unidad Iztapalapa. Bases de datos SNIB-CONABIO proyecto No. J121. México, D.F.

Ramírez-Pulido, J. 2007. Diversidad de los mamíferos de la Reserva de la biósfera Tehuacán-Cuicatlán, Puebla-Oaxaca, México. Universidad Autónoma Metropolitana-Unidad Iztapalapa. Bases de datos SNIB-CONABIO proyecto No. BK022. México, D.F.

Rentail, N. P. 2004. UAZ Vertebrates collections at the University of Arizona, database of Mexican specimens. The University of Arizona. Tucson, Arizona. Bases de datos SNIB-CONABIO. México.

Ressl, R., Cruz-López, M. I., Lara-Morales, L., Gual-Díaz, M., Ocaña-Nava, D., Díaz-Maeda, P. G., Pérez-Bello, A., Wickel, A. J. 2008. Sistema de información sobre Bosque Mesófilo de Montaña de México para apoyo en programas de restauración (Fase 1). Comisión Nacional para el Conocimiento y Uso de la Biodiversidad. Bases de datos SNIB-CONABIO proyecto No. EQ007. México, D.F.

Sam Noble Oklahoma Museum of Natural History, Mammals Specimens (accessed through GBIF data portal, https://www.gbif.org).

Sánchez-Cordero, V. 2006. Estado actual del conocimiento biológico de algunas especies de roedores de las familias Muridae, Geomyidae, Heteromyidae y Sciuridae (Rodentia: Mammalia) incluidas en el PROY-NOM-059-ECOL-2000. Instituto de Biología. Bases de datos SNIB-CONABIO proyectos No. W036, L047 y A026. México, D.F.

Servín-Martínez, J. I. 1998. Los mamí­feros del estado de Durango, México. Instituto de Ecologí A.C. Centro Regional-Durango. Bases de datos SNIB-CONABIO proyecto No. P064. México, D.F.

Sosa-Fernández, V., Hernández A., Bello, J. 1998. Inventario de los mamíferos de las reservas de la biósfera Mapimí, La Michilía, El Cielo y Calakmul. Instituto de Ecología A.C. Bases de datos SNIB-CONABIO proyecto No. P027. México, D.F.

TTU Mammals Collection (accessed through SNIB CONABIO).

UCLA-Dickey Collection (UCLA-Dickey), Bird and Mammal specimens (accessed through GBIF data portal, https://www.gbif.org).

UMMZ Mammal Collection (accessed through SNIB CONABIO).

UNIBIO, IBUNAM, CNMA/Colección Nacional de mamíferos (accessed through GBIF data portal, https://www.gbif.org).

University of Kansas Biodiversity Institute, KUBI Mammalogy Collection (accessed through GBIF data portal, https://www.gbif.org).

University of Michigan Museum of Zoology, Mammal specimens (accessed through GBIF data portal, https://www.gbif.org).

University of Minnesota Bell Museum of Natural History, Mammal specimens (accessed through GBIF data portal, https://www.gbif.org).

Vargas-Cuenca. J., Hortelano-Mocanda, Y., Cervantes, F. A. 2014. Actualización de la base de datos del estado de Morelos de la Colección Nacional de Mamíferos del Instituto de Biología, UNAM. Universidad Nacional Autónoma de México. Instituto de Biología. Bases de datos SNIB-CONABIO proyecto No. JL003, HA023 y V043. México, D.F.

Velázquez, J. A., Romero-Malpica, F. J.1998. Análisis de la heterogeneidad ambiental y conectividad de las Áreas naturales del sur del Valle de México. Universidad Nacional Autónoma de México. Facultad de Ciencias. Bases de datos SNIB-CONABIO proyecto No. B144. México, D.F.

Velázquez, J. A., Bocco, G. 2004. Biodiversidad, hábitat y manejo en el Parque Nacional Pico de Tancítaro, Michoacán, México. Universidad Nacional Autónoma de México. Instituto de Geografía. Bases de datos SNIB-CONABIO proyecto No. R092. México, D.F.

Table S3. Bioclimatic variables selected for each species for building ecological niche models.

| Species | Bioclimatic variables |
| --- | --- |
| Callospermophilus madrensis | 1,2,4,5,9,12,15,17,19 |
| Chaetodipus arerius | 1,2,4,5,6,8,12,15,17,19 |
| Chaetodipus artus | 1,2,4,5,9,12,15,17,19 |
| Chaetodipus goldmani | 1,2,4,5,8,9,12,15,17,19 |
| Chaetodipus pernix | 1,2,3,8,15,17,19 |
| Cratogeomys fumosus | 1,2,3,4,5,12,15,17,18,19 |
| Cratogeomys goldmani | 1,2,3,4,5,9,12,15,17,18,19 |
| Cratogeomys merriami | 1,2,3,7,12,15,16,17,18,19 |
| Cynomys mexicanus | 1,2,3,5,9,12,15,17,19 |
| Dasyprocta mexicana | 1,2,3,4,12,15,17 |
| Dipodomys nelsoni | 1,2,3,5,7,9,12,15,17,19 |
| Dipodomys ornatus | 1,2,3,5,7,9,12,15,17,19 |
| Dipodomys phillipsii | 1,2,4,5,6,9,12,13,17,19 |
| Megadontomys cryophilus | 1,2,3,4,7,12,15,19 |
| Microtus oaxacensis | 1,2,3,4,7,12 |
| Microtus quasiater | 1,2,3,4,7,12,19 |
| Neotamias durangae | 1,2,3,4,5,9,12,15,17,19 |
| Neotoma goldmani | 1,2,4,5,12,15,17,19 |
| Neotomodon alstoni | 1,3,4,7,12,15,17,18 |
| Notocitellus adocetus | 1,3,7,12,15,17,18,19 |
| Notocitellus annulatus | 1,2,3,7,12,15,17,18,19 |
| Oryzomys albiventer | 1,3,7,12,15,17,18,19 |
| Oryzomys chapmani | 1,2,7,12,15 |
| Oryzomys guerrerensis | 1,3,4,7,12 |
| Oryzomys melanotis | 1,3,4,7,12,15,17,18,19 |
| Osgoodomys banderanus | 1,3,7,12,15,17,18,19 |
| Pappogeomys bulleri | 1,3,7,12,15,17,18,19 |
| Peromyscus difficilis | 1,2,4,5,8,9,12,15,17 |
| Peromyscus eva | 1,2,4,5,6,12,15,17,19 |
| Peromyscus furvus | 1,2,3,4,7,12,15,19 |
| Peromyscus hylocetes | 1,2,3,7,12,15,17,19 |
| Peromyscus levipes | 1,2,3,6,7,9,12,15,17 |
| Peromyscus megalops | 1,2,3,4,12,15 |
| Peromyscus melanocarpus | 1,2,3,4,7,12,15,17 |
| Peromyscus melanophrys | 1,2,3,5,7,8,9,12,18,19 |
| Peromyscus perfulvus | 1,2,3,4,12,15,17,18 |
| Peromyscus zarhynchus | 1,3,6,7,12,13,17,19 |
| Reithrodontomys chrysopsis | 1,2,3,4,12,15,18 |
| Sciurus alleni | 1,2,3,5,7,9,12,15,17,19 |
| Sciurus colliaei | 1,2,5,7,8,9,12,15,17,19 |
| Sigmodon alleni | 1,2,7,9,12,15,18,19 |
| Sigmodon leucotis | 1,2,3,4,5,12,13,15 |
| Sigmodon mascotensis | 1,3,7,12,15,18,19 |
| Thomomys atrovarius | 1,2,3,7,12,15,17,19 |

Table S4. Results of the binomial test for the calibration models for the algorithms used: Bioclim, Generalized Additive Models (GAM), Generalized Linear Models (GLM), Boosted Regression Trees (BRT), Maxent, Random Forest (RF), and Support Vector Machine (SVM) across two time periods, T1 (1950-1979) and T2 (1980-2009).

| Species | Binomial test | p | Occ success | Occ total | Algorithm | Period |
| --- | --- | --- | --- | --- | --- | --- |
| Callospermophilus madrensis | 0.0038 | 5.53E-07 | 2 | 5 | Bioclim | T1 |
| Callospermophilus madrensis | 0.0162 | 1.69E-05 | 2 | 4 | Bioclim | T2 |
| Callospermophilus madrensis | 0.0256 | 2.11E-06 | 3 | 5 | BRT | T1 |
| Callospermophilus madrensis | 0.0257 | 0.00E+00 | 4 | 4 | BRT | T2 |
| Callospermophilus madrensis | 0.0348 | 3.99E-04 | 2 | 5 | GAM | T1 |
| Callospermophilus madrensis | 0.0495 | 1.37E-02 | 1 | 4 | GAM | T2 |
| Callospermophilus madrensis | 0.492 | 9.66E-01 | 0 | 5 | GLM | T1 |
| Callospermophilus madrensis | 0.0552 | 1.69E-02 | 1 | 4 | GLM | T2 |
| Callospermophilus madrensis | 0.0368 | 0.00E+00 | 5 | 5 | Maxent | T1 |
| Callospermophilus madrensis | 0.008 | 0.00E+00 | 4 | 4 | Maxent | T2 |
| Callospermophilus madrensis | 0.011 | 7.30E-08 | 3 | 5 | RF | T1 |
| Callospermophilus madrensis | 0.0182 | 0.00E+00 | 4 | 4 | RF | T2 |
| Callospermophilus madrensis | 0.0164 | 3.55E-07 | 3 | 5 | SVM | T1 |
| Callospermophilus madrensis | 0.0284 | 0.00E+00 | 4 | 4 | SVM | T2 |
| Chaetodipus arenarius | 0.2518 | 1.26E-05 | 13 | 19 | Bioclim | T1 |
| Chaetodipus arenarius | 0.2606 | 0.00E+00 | 49 | 54 | Bioclim | T2 |
| Chaetodipus arenarius | 0.259 | 7.13E-12 | 18 | 19 | BRT | T1 |
| Chaetodipus arenarius | 0.1975 | 0.00E+00 | 50 | 54 | BRT | T2 |
| Chaetodipus arenarius | 0.0933 | 2.78E-11 | 13 | 19 | GAM | T1 |
| Chaetodipus arenarius | 0.132 | 0.00E+00 | 46 | 54 | GAM | T2 |
| Chaetodipus arenarius | 0.1602 | 2.38E-09 | 14 | 19 | GLM | T1 |
| Chaetodipus arenarius | 0.1907 | 0.00E+00 | 51 | 54 | GLM | T2 |
| Chaetodipus arenarius | 0.2511 | 2.28E-10 | 17 | 19 | Maxent | T1 |
| Chaetodipus arenarius | 0.1397 | 0.00E+00 | 47 | 54 | Maxent | T2 |
| Chaetodipus arenarius | 0.0603 | 7.87E-11 | 11 | 19 | RF | T1 |
| Chaetodipus arenarius | 0.0662 | 0.00E+00 | 33 | 54 | RF | T2 |
| Chaetodipus arenarius | 0.1686 | 4.91E-09 | 14 | 19 | SVM | T1 |
| Chaetodipus arenarius | 0.1713 | 0.00E+00 | 47 | 54 | SVM | T2 |
| Chaetodipus artus | 0.0601 | 0.00E+00 | 17 | 20 | Bioclim | T1 |
| Chaetodipus artus | 0.0614 | 4.02E-03 | 2 | 6 | Bioclim | T2 |
| Chaetodipus artus | 0.081 | 0.00E+00 | 19 | 20 | BRT | T1 |
| Chaetodipus artus | 0.1877 | 0.00E+00 | 6 | 6 | BRT | T2 |
| Chaetodipus artus | 0.0303 | 0.00E+00 | 16 | 20 | GAM | T1 |
| Chaetodipus artus | 0.0336 | 7.01E-04 | 2 | 6 | GAM | T2 |
| Chaetodipus artus | 0.0653 | 0.00E+00 | 18 | 20 | GLM | T1 |
| Chaetodipus artus | 0.1186 | 1.27E-04 | 4 | 6 | GLM | T2 |
| Chaetodipus artus | 0.0422 | 0.00E+00 | 19 | 20 | Maxent | T1 |
| Chaetodipus artus | 0.2083 | 1.94E-03 | 4 | 6 | Maxent | T2 |
| Chaetodipus artus | 0.0348 | 0.00E+00 | 16 | 20 | RF | T1 |
| Chaetodipus artus | 0.07 | 3.22E-04 | 3 | 6 | RF | T2 |
| Chaetodipus artus | 0.0672 | 0.00E+00 | 19 | 20 | SVM | T1 |
| Chaetodipus artus | 0.1655 | 0.00E+00 | 6 | 6 | SVM | T2 |
| Chaetodipus goldmani | 0.0692 | 0.00E+00 | 32 | 38 | Bioclim | T1 |
| Chaetodipus goldmani | 0.1114 | 3.13E-06 | 8 | 14 | Bioclim | T2 |
| Chaetodipus goldmani | 0.217 | 0.00E+00 | 33 | 38 | BRT | T1 |
| Chaetodipus goldmani | 0.1127 | 9.81E-09 | 10 | 14 | BRT | T2 |
| Chaetodipus goldmani | 0.0248 | 0.00E+00 | 29 | 38 | GAM | T1 |
| Chaetodipus goldmani | 0.0509 | 1.89E-12 | 10 | 14 | GAM | T2 |
| Chaetodipus goldmani | 0.0728 | 0.00E+00 | 35 | 38 | GLM | T1 |
| Chaetodipus goldmani | 0.0943 | 3.76E-11 | 11 | 14 | GLM | T2 |
| Chaetodipus goldmani | 0.0577 | 0.00E+00 | 35 | 38 | Maxent | T1 |
| Chaetodipus goldmani | 0.0868 | 1.75E-08 | 9 | 14 | Maxent | T2 |
| Chaetodipus goldmani | 0.0265 | 0.00E+00 | 32 | 38 | RF | T1 |
| Chaetodipus goldmani | 0.0419 | 2.24E-13 | 10 | 14 | RF | T2 |
| Chaetodipus goldmani | 0.0595 | 0.00E+00 | 35 | 38 | SVM | T1 |
| Chaetodipus goldmani | 0.097 | 1.97E-09 | 10 | 14 | SVM | T2 |
| Chaetodipus pernix | 0.1435 | 0.00E+00 | 32 | 36 | Bioclim | T1 |
| Chaetodipus pernix | 0.3468 | 8.74E-06 | 10 | 11 | Bioclim | T2 |
| Chaetodipus pernix | 0.151 | 0.00E+00 | 29 | 36 | BRT | T1 |
| Chaetodipus pernix | 0.366 | 0.00E+00 | 11 | 11 | BRT | T2 |
| Chaetodipus pernix | 0.0965 | 0.00E+00 | 30 | 36 | GAM | T1 |
| Chaetodipus pernix | 0.1962 | 2.04E-04 | 7 | 11 | GAM | T2 |
| Chaetodipus pernix | 0.1189 | 0.00E+00 | 31 | 36 | GLM | T1 |
| Chaetodipus pernix | 0.2119 | 2.80E-03 | 6 | 11 | GLM | T2 |
| Chaetodipus pernix | 0.1003 | 0.00E+00 | 31 | 36 | Maxent | T1 |
| Chaetodipus pernix | 0.2621 | 1.28E-05 | 9 | 11 | Maxent | T2 |
| Chaetodipus pernix | 0.082 | 0.00E+00 | 28 | 36 | RF | T1 |
| Chaetodipus pernix | 0.162 | 3.09E-06 | 8 | 11 | RF | T2 |
| Chaetodipus pernix | 0.0973 | 0.00E+00 | 28 | 36 | SVM | T1 |
| Chaetodipus pernix | 0.2733 | 6.35E-07 | 10 | 11 | SVM | T2 |
| Cratogeomys goldmani | 0.1016 | 1.19E-11 | 10 | 11 | Bioclim | T1 |
| Cratogeomys goldmani | 0.1057 | 5.74E-07 | 7 | 10 | Bioclim | T2 |
| Cratogeomys goldmani | 0.284 | 9.68E-07 | 10 | 11 | BRT | T1 |
| Cratogeomys goldmani | 0.157 | 4.98E-07 | 8 | 10 | BRT | T2 |
| Cratogeomys goldmani | 0.037 | 1.02E-06 | 5 | 11 | GAM | T1 |
| Cratogeomys goldmani | 0.0461 | 8.39E-10 | 7 | 10 | GAM | T2 |
| Cratogeomys goldmani | 0.1111 | 2.83E-09 | 9 | 11 | GLM | T1 |
| Cratogeomys goldmani | 0.1232 | 8.03E-10 | 9 | 10 | GLM | T2 |
| Cratogeomys goldmani | 0.1055 | 1.80E-11 | 10 | 11 | Maxent | T1 |
| Cratogeomys goldmani | 0.1299 | 1.37E-09 | 9 | 10 | Maxent | T2 |
| Cratogeomys goldmani | 0.028 | 5.75E-11 | 7 | 11 | RF | T1 |
| Cratogeomys goldmani | 0.0062 | 4.13E-14 | 6 | 10 | RF | T2 |
| Cratogeomys goldmani | 0.1721 | 0.00E+00 | 11 | 11 | SVM | T1 |
| Cratogeomys goldmani | 0.1116 | 8.78E-07 | 7 | 10 | SVM | T2 |
| Cratogeomys merriami | 0.0379 | 3.18E-11 | 8 | 12 | Bioclim | T1 |
| Cratogeomys merriami | 0.0757 | 3.90E-09 | 8 | 11 | Bioclim | T2 |
| Cratogeomys merriami | 0.0934 | 4.39E-13 | 11 | 12 | BRT | T1 |
| Cratogeomys merriami | 0.2109 | 1.55E-06 | 9 | 11 | BRT | T2 |
| Cratogeomys merriami | 0.0458 | 1.72E-10 | 8 | 12 | GAM | T1 |
| Cratogeomys merriami | 0.0325 | 1.13E-08 | 6 | 11 | GAM | T2 |
| Cratogeomys merriami | 0.0903 | 2.95E-13 | 11 | 12 | GLM | T1 |
| Cratogeomys merriami | 0.0621 | 3.09E-08 | 7 | 11 | GLM | T2 |
| Cratogeomys merriami | 0.0497 | 5.24E-14 | 10 | 12 | Maxent | T1 |
| Cratogeomys merriami | 0.0452 | 4.00E-11 | 8 | 11 | Maxent | T2 |
| Cratogeomys merriami | 0.0151 | 1.00E-08 | 5 | 12 | RF | T1 |
| Cratogeomys merriami | 0.0181 | 1.48E-08 | 5 | 11 | RF | T2 |
| Cratogeomys merriami | 0.0576 | 1.28E-06 | 6 | 12 | SVM | T1 |
| Cratogeomys merriami | 0.0538 | 1.01E-08 | 7 | 11 | SVM | T2 |
| Cynomys mexicanus | 0.0344 | 1.12E-08 | 5 | 7 | BRT | T1 |
| Cynomys mexicanus | 0.0497 | 0.00E+00 | 96 | 122 | BRT | T2 |
| Cynomys mexicanus | 0.012 | 2.05E-11 | 5 | 7 | GAM | T1 |
| Cynomys mexicanus | 0.0062 | 0.00E+00 | 106 | 122 | GAM | T2 |
| Cynomys mexicanus | 0.0246 | 1.52E-09 | 5 | 7 | GLM | T1 |
| Cynomys mexicanus | 0.0098 | 0.00E+00 | 110 | 122 | GLM | T2 |
| Cynomys mexicanus | 0.0097 | 3.14E-05 | 2 | 7 | RF | T1 |
| Cynomys mexicanus | 0.0029 | 0.00E+00 | 105 | 122 | RF | T2 |
| Cynomys mexicanus | 0.0129 | 3.12E-11 | 5 | 7 | SVM | T1 |
| Cynomys mexicanus | 0.009 | 0.00E+00 | 109 | 122 | SVM | T2 |
| Dasyprocta mexicana | 0.0106 | 1.10E-03 | 1 | 5 | Bioclim | T1 |
| Dasyprocta mexicana | 0.0141 | 5.87E-04 | 1 | 3 | Bioclim | T2 |
| Dasyprocta mexicana | 0.0338 | 6.37E-06 | 3 | 5 | BRT | T1 |
| Dasyprocta mexicana | 0.1668 | 4.64E-03 | 2 | 3 | BRT | T2 |
| Dasyprocta mexicana | 0.0238 | 1.59E-06 | 3 | 5 | GAM | T1 |
| Dasyprocta mexicana | 0.0987 | 2.73E-02 | 1 | 3 | GAM | T2 |
| Dasyprocta mexicana | 0.4894 | 9.65E-01 | 0 | 5 | GLM | T1 |
| Dasyprocta mexicana | 0.2261 | 1.16E-02 | 2 | 3 | GLM | T2 |
| Dasyprocta mexicana | 0.0352 | 1.15E-02 | 1 | 5 | Maxent | T1 |
| Dasyprocta mexicana | 0.4931 | 1.20E-01 | 2 | 3 | Maxent | T2 |
| Dasyprocta mexicana | 0.023 | 6.43E-09 | 4 | 5 | RF | T1 |
| Dasyprocta mexicana | 0.1033 | 2.98E-02 | 1 | 3 | RF | T2 |
| Dasyprocta mexicana | 0.0244 | 1.74E-06 | 3 | 5 | SVM | T1 |
| Dasyprocta mexicana | 0.0666 | 1.27E-02 | 1 | 3 | SVM | T2 |
| Dipodomys nelsoni | 0.214 | 0.00E+00 | 30 | 33 | Bioclim | T1 |
| Dipodomys nelsoni | 0.1925 | 4.95E-11 | 15 | 17 | Bioclim | T2 |
| Dipodomys nelsoni | 0.2871 | 4.44E-15 | 30 | 33 | BRT | T1 |
| Dipodomys nelsoni | 0.3262 | 5.36E-09 | 16 | 17 | BRT | T2 |
| Dipodomys nelsoni | 0.1124 | 0.00E+00 | 26 | 33 | GAM | T1 |
| Dipodomys nelsoni | 0.0788 | 0.00E+00 | 15 | 17 | GAM | T2 |
| Dipodomys nelsoni | 0.1666 | 0.00E+00 | 29 | 33 | GLM | T1 |
| Dipodomys nelsoni | 0.2183 | 5.79E-12 | 16 | 17 | GLM | T2 |
| Dipodomys nelsoni | 0.1737 | 0.00E+00 | 30 | 33 | Maxent | T1 |
| Dipodomys nelsoni | 0.1923 | 6.75E-13 | 16 | 17 | Maxent | T2 |
| Dipodomys nelsoni | 0.0326 | 0.00E+00 | 17 | 33 | RF | T1 |
| Dipodomys nelsoni | 0.0354 | 2.02E-14 | 11 | 17 | RF | T2 |
| Dipodomys nelsoni | 0.1536 | 1.77E-14 | 24 | 33 | SVM | T1 |
| Dipodomys nelsoni | 0.2083 | 0.00E+00 | 17 | 17 | SVM | T2 |
| Dipodomys ornatus | 0.0332 | 7.73E-09 | 7 | 15 | Bioclim | T1 |
| Dipodomys ornatus | 0.0442 | 3.30E-06 | 4 | 7 | Bioclim | T2 |
| Dipodomys ornatus | 0.1393 | 1.44E-13 | 14 | 15 | BRT | T1 |
| Dipodomys ornatus | 0.1201 | 1.88E-05 | 5 | 7 | BRT | T2 |
| Dipodomys ornatus | 0.03 | 8.40E-11 | 8 | 15 | GAM | T1 |
| Dipodomys ornatus | 0.0627 | 4.64E-04 | 3 | 7 | GAM | T2 |
| Dipodomys ornatus | 0.1081 | 4.00E-13 | 13 | 15 | GLM | T1 |
| Dipodomys ornatus | 0.1297 | 2.96E-05 | 5 | 7 | GLM | T2 |
| Dipodomys ornatus | 0.0532 | 2.66E-15 | 12 | 15 | Maxent | T1 |
| Dipodomys ornatus | 0.1278 | 5.72E-04 | 4 | 7 | Maxent | T2 |
| Dipodomys ornatus | 0.0264 | 1.29E-09 | 7 | 15 | RF | T1 |
| Dipodomys ornatus | 0.016 | 2.23E-06 | 3 | 7 | RF | T2 |
| Dipodomys ornatus | 0.0461 | 3.67E-09 | 8 | 15 | SVM | T1 |
| Dipodomys ornatus | 0.0847 | 7.93E-05 | 4 | 7 | SVM | T2 |
| Dipodomys phillipsii | 0.0109 | 3.53E-04 | 1 | 3 | Bioclim | T1 |
| Dipodomys phillipsii | 0.01 | 5.83E-02 | 0 | 6 | Bioclim | T2 |
| Dipodomys phillipsii | 0.068 | 2.87E-04 | 3 | 6 | BRT | T2 |
| Dipodomys phillipsii | 0.0479 | 1.10E-04 | 2 | 3 | GAM | T1 |
| Dipodomys phillipsii | 0.0718 | 3.55E-04 | 3 | 6 | GAM | T2 |
| Dipodomys phillipsii | 0.6357 | 9.52E-01 | 0 | 3 | GLM | T1 |
| Dipodomys phillipsii | 0.6357 | 9.98E-01 | 0 | 6 | GLM | T2 |
| Dipodomys phillipsii | 0.0733 | 3.94E-04 | 2 | 3 | Maxent | T1 |
| Dipodomys phillipsii | 0.0286 | 1.13E-07 | 4 | 6 | Maxent | T2 |
| Dipodomys phillipsii | 0.0106 | 1.21E-06 | 2 | 3 | RF | T1 |
| Dipodomys phillipsii | 0.0083 | 1.11E-05 | 2 | 6 | RF | T2 |
| Dipodomys phillipsii | 0.0655 | 0.00E+00 | 3 | 3 | SVM | T1 |
| Dipodomys phillipsii | 0.0386 | 0.00E+00 | 6 | 6 | SVM | T2 |
| Megadontomys cryophilus | 0.0077 | 2.74E-11 | 4 | 5 | Bioclim | T1 |
| Megadontomys cryophilus | 0.0103 | 6.94E-10 | 4 | 6 | Bioclim | T2 |
| Megadontomys cryophilus | 0.1576 | 0.00E+00 | 5 | 5 | BRT | T1 |
| Megadontomys cryophilus | 0.1677 | 2.23E-05 | 5 | 6 | BRT | T2 |
| Megadontomys cryophilus | 0.0164 | 1.20E-09 | 4 | 5 | GAM | T1 |
| Megadontomys cryophilus | 0.048 | 7.35E-05 | 3 | 6 | GAM | T2 |
| Megadontomys cryophilus | 0.1164 | 0.00E+00 | 5 | 5 | GLM | T1 |
| Megadontomys cryophilus | 0.0271 | 0.00E+00 | 6 | 6 | GLM | T2 |
| Megadontomys cryophilus | 0.0447 | 0.00E+00 | 5 | 5 | Maxent | T1 |
| Megadontomys cryophilus | 0.0119 | 0.00E+00 | 6 | 6 | Maxent | T2 |
| Megadontomys cryophilus | 0.0103 | 1.14E-10 | 4 | 5 | RF | T1 |
| Megadontomys cryophilus | 0.0255 | 0.00E+00 | 6 | 6 | RF | T2 |
| Megadontomys cryophilus | 0.0388 | 0.00E+00 | 5 | 5 | SVM | T1 |
| Megadontomys cryophilus | 0.0487 | 0.00E+00 | 6 | 6 | SVM | T2 |
| Microtus oaxacensis | 0.002 | 1.21E-05 | 1 | 3 | Bioclim | T1 |
| Microtus oaxacensis | 0.0009 | 7.59E-13 | 3 | 4 | Bioclim | T2 |
| Microtus oaxacensis | 0.0263 | 4.80E-07 | 3 | 4 | BRT | T2 |
| Microtus oaxacensis | 0.2262 | 1.16E-02 | 2 | 3 | GAM | T1 |
| Microtus oaxacensis | 0.043 | 3.07E-04 | 2 | 4 | GAM | T2 |
| Microtus oaxacensis | 0.1406 | 2.78E-03 | 2 | 3 | GLM | T1 |
| Microtus oaxacensis | 0.0704 | 2.46E-05 | 3 | 4 | GLM | T2 |
| Microtus oaxacensis | 0.06 | 0.00E+00 | 3 | 3 | Maxent | T1 |
| Microtus oaxacensis | 0.0046 | 4.29E-10 | 3 | 4 | Maxent | T2 |
| Microtus oaxacensis | 0.0066 | 2.86E-07 | 2 | 3 | RF | T1 |
| Microtus oaxacensis | 0.0153 | 5.44E-08 | 3 | 4 | RF | T2 |
| Microtus oaxacensis | 0.0132 | 2.29E-06 | 2 | 3 | SVM | T1 |
| Microtus oaxacensis | 0.0066 | 1.89E-09 | 3 | 4 | SVM | T2 |
| Microtus quasiater | 0.0123 | 2.76E-10 | 4 | 5 | Bioclim | T1 |
| Microtus quasiater | 0.0732 | 0.00E+00 | 16 | 18 | Bioclim | T2 |
| Microtus quasiater | 0.0484 | 0.00E+00 | 5 | 5 | BRT | T1 |
| Microtus quasiater | 0.5944 | 1.14E-03 | 16 | 18 | BRT | T2 |
| Microtus quasiater | 0.0493 | 2.20E-02 | 1 | 5 | GAM | T1 |
| Microtus quasiater | 0.0421 | 0.00E+00 | 15 | 18 | GAM | T2 |
| Microtus quasiater | 0.0894 | 0.00E+00 | 5 | 5 | GLM | T1 |
| Microtus quasiater | 0.1514 | 1.78E-15 | 17 | 18 | GLM | T2 |
| Microtus quasiater | 0.0435 | 0.00E+00 | 5 | 5 | Maxent | T1 |
| Microtus quasiater | 0.048 | 0.00E+00 | 16 | 18 | Maxent | T2 |
| Microtus quasiater | 0.0086 | 4.61E-11 | 4 | 5 | RF | T1 |
| Microtus quasiater | 0.039 | 0.00E+00 | 16 | 18 | RF | T2 |
| Microtus quasiater | 0.0189 | 0.00E+00 | 5 | 5 | SVM | T1 |
| Microtus quasiater | 0.0724 | 0.00E+00 | 17 | 18 | SVM | T2 |
| Neotamias durangae | 0.0313 | 4.03E-10 | 7 | 12 | Bioclim | T1 |
| Neotamias durangae | 0.0133 | 3.93E-02 | 0 | 3 | Bioclim | T2 |
| Neotamias durangae | 0.1025 | 6.97E-09 | 9 | 12 | BRT | T1 |
| Neotamias durangae | 0.0539 | 1.56E-04 | 2 | 3 | BRT | T2 |
| Neotamias durangae | 0.0416 | 3.87E-06 | 5 | 12 | GAM | T1 |
| Neotamias durangae | 0.072 | 3.73E-04 | 2 | 3 | GAM | T2 |
| Neotamias durangae | 0.0374 | 6.88E-08 | 6 | 12 | GLM | T1 |
| Neotamias durangae | 0.5164 | 8.87E-01 | 0 | 3 | GLM | T2 |
| Neotamias durangae | 0.0407 | 7.54E-13 | 9 | 12 | Maxent | T1 |
| Neotamias durangae | 0.2914 | 0.00E+00 | 3 | 3 | Maxent | T2 |
| Neotamias durangae | 0.0296 | 2.64E-10 | 7 | 12 | RF | T1 |
| Neotamias durangae | 0.0334 | 3.74E-05 | 2 | 3 | RF | T2 |
| Neotamias durangae | 0.0334 | 1.06E-13 | 9 | 12 | SVM | T1 |
| Neotamias durangae | 0.054 | 1.58E-04 | 2 | 3 | SVM | T2 |
| Neotoma goldmani | 0.1103 | 1.99E-07 | 6 | 7 | Bioclim | T1 |
| Neotoma goldmani | 0.0256 | 0.00E+00 | 3 | 3 | Bioclim | T2 |
| Neotoma goldmani | 0.0992 | 6.10E-06 | 5 | 7 | BRT | T1 |
| Neotoma goldmani | 0.085 | 0.00E+00 | 3 | 3 | BRT | T2 |
| Neotoma goldmani | 0.0608 | 3.37E-07 | 5 | 7 | GAM | T1 |
| Neotoma goldmani | 0.0944 | 0.00E+00 | 3 | 3 | GAM | T2 |
| Neotoma goldmani | 0.1283 | 5.83E-04 | 4 | 7 | GLM | T1 |
| Neotoma goldmani | 0.2176 | 5.21E-01 | 0 | 3 | GLM | T2 |
| Neotoma goldmani | 0.1594 | 9.92E-05 | 5 | 7 | Maxent | T1 |
| Neotoma goldmani | 0.0484 | 0.00E+00 | 3 | 3 | Maxent | T2 |
| Neotoma goldmani | 0.0312 | 5.90E-07 | 4 | 7 | RF | T1 |
| Neotoma goldmani | 0.0266 | 2.09E-03 | 1 | 3 | RF | T2 |
| Neotoma goldmani | 0.1237 | 4.89E-04 | 4 | 7 | SVM | T1 |
| Neotoma goldmani | 0.0619 | 0.00E+00 | 3 | 3 | SVM | T2 |
| Neotomodon alstoni | 0.1391 | 0.00E+00 | 25 | 26 | Bioclim | T1 |
| Neotomodon alstoni | 0.1158 | 0.00E+00 | 30 | 36 | Bioclim | T2 |
| Neotomodon alstoni | 0.0934 | 0.00E+00 | 23 | 26 | BRT | T1 |
| Neotomodon alstoni | 0.0831 | 0.00E+00 | 33 | 36 | BRT | T2 |
| Neotomodon alstoni | 0.022 | 0.00E+00 | 22 | 26 | GAM | T1 |
| Neotomodon alstoni | 0.0412 | 0.00E+00 | 34 | 36 | GAM | T2 |
| Neotomodon alstoni | 0.0777 | 0.00E+00 | 23 | 26 | GLM | T1 |
| Neotomodon alstoni | 0.0582 | 0.00E+00 | 33 | 36 | GLM | T2 |
| Neotomodon alstoni | 0.0349 | 0.00E+00 | 22 | 26 | Maxent | T1 |
| Neotomodon alstoni | 0.0521 | 0.00E+00 | 33 | 36 | Maxent | T2 |
| Neotomodon alstoni | 0.0243 | 0.00E+00 | 23 | 26 | RF | T1 |
| Neotomodon alstoni | 0.0251 | 0.00E+00 | 29 | 36 | RF | T2 |
| Neotomodon alstoni | 0.0889 | 0.00E+00 | 21 | 26 | SVM | T1 |
| Neotomodon alstoni | 0.0739 | 0.00E+00 | 33 | 36 | SVM | T2 |
| Notocitellus adocetus | 0.0922 | 4.30E-02 | 2 | 9 | Bioclim | T1 |
| Notocitellus adocetus | 0.1142 | 3.65E-02 | 2 | 7 | Bioclim | T2 |
| Notocitellus adocetus | 0.1264 | 1.47E-05 | 6 | 9 | BRT | T1 |
| Notocitellus adocetus | 0.2597 | 1.67E-03 | 5 | 7 | BRT | T2 |
| Notocitellus adocetus | 0.0987 | 8.38E-04 | 4 | 9 | GAM | T1 |
| Notocitellus adocetus | 0.1791 | 2.80E-03 | 4 | 7 | GAM | T2 |
| Notocitellus adocetus | 0.1014 | 6.97E-05 | 5 | 9 | GLM | T1 |
| Notocitellus adocetus | 0.1701 | 4.12E-06 | 6 | 7 | GLM | T2 |
| Notocitellus adocetus | 0.1252 | 1.37E-05 | 6 | 9 | Maxent | T1 |
| Notocitellus adocetus | 0.1084 | 1.03E-05 | 5 | 7 | Maxent | T2 |
| Notocitellus adocetus | 0.0537 | 1.75E-06 | 5 | 9 | RF | T1 |
| Notocitellus adocetus | 0.0736 | 1.04E-06 | 5 | 7 | RF | T2 |
| Notocitellus adocetus | 0.0682 | 1.48E-04 | 4 | 9 | SVM | T1 |
| Notocitellus adocetus | 0.1053 | 1.43E-07 | 6 | 7 | SVM | T2 |
| Notocitellus annulatus | 0.0398 | 7.94E-05 | 3 | 7 | Bioclim | T1 |
| Notocitellus annulatus | 0.0376 | 4.37E-07 | 4 | 6 | Bioclim | T2 |
| Notocitellus annulatus | 0.2697 | 0.00E+00 | 6 | 6 | BRT | T2 |
| Notocitellus annulatus | 0.0702 | 3.16E-05 | 4 | 7 | GAM | T1 |
| Notocitellus annulatus | 0.179 | 1.13E-02 | 3 | 6 | GAM | T2 |
| Notocitellus annulatus | 0.1124 | 3.09E-04 | 4 | 7 | GLM | T1 |
| Notocitellus annulatus | 0.1889 | 1.22E-03 | 4 | 6 | GLM | T2 |
| Notocitellus annulatus | 0.1002 | 6.46E-06 | 5 | 7 | Maxent | T1 |
| Notocitellus annulatus | 0.2091 | 1.98E-03 | 4 | 6 | Maxent | T2 |
| Notocitellus annulatus | 0.0404 | 8.48E-05 | 3 | 7 | RF | T1 |
| Notocitellus annulatus | 0.0702 | 1.20E-07 | 5 | 6 | RF | T2 |
| Notocitellus annulatus | 0.0711 | 3.38E-05 | 4 | 7 | SVM | T1 |
| Notocitellus annulatus | 0.0818 | 0.00E+00 | 6 | 6 | SVM | T2 |
| Oryzomys albiventer | 0.0922 | 1.91E-10 | 12 | 18 | Bioclim | T1 |
| Oryzomys albiventer | 0.1636 | 3.75E-14 | 20 | 24 | Bioclim | T2 |
| Oryzomys albiventer | 0.2647 | 4.13E-04 | 11 | 18 | BRT | T1 |
| Oryzomys albiventer | 0.2529 | 2.56E-10 | 20 | 24 | BRT | T2 |
| Oryzomys albiventer | 0.0745 | 1.85E-06 | 8 | 18 | GAM | T1 |
| Oryzomys albiventer | 0.0858 | 0.00E+00 | 22 | 24 | GAM | T2 |
| Oryzomys albiventer | 0.1839 | 9.26E-07 | 12 | 18 | GLM | T1 |
| Oryzomys albiventer | 0.2444 | 1.29E-10 | 20 | 24 | GLM | T2 |
| Oryzomys albiventer | 0.2394 | 1.85E-07 | 14 | 18 | Maxent | T1 |
| Oryzomys albiventer | 0.1272 | 0.00E+00 | 22 | 24 | Maxent | T2 |
| Oryzomys albiventer | 0.0779 | 1.99E-07 | 9 | 18 | RF | T1 |
| Oryzomys albiventer | 0.045 | 0.00E+00 | 17 | 24 | RF | T2 |
| Oryzomys albiventer | 0.1355 | 3.19E-07 | 11 | 18 | SVM | T1 |
| Oryzomys albiventer | 0.1451 | 0.00E+00 | 22 | 24 | SVM | T2 |
| Oryzomys chapmani | 0.068 | 0.00E+00 | 15 | 15 | Bioclim | T1 |
| Oryzomys chapmani | 0.2045 | 0.00E+00 | 39 | 47 | Bioclim | T2 |
| Oryzomys chapmani | 0.2438 | 6.39E-10 | 14 | 15 | BRT | T1 |
| Oryzomys chapmani | 0.4195 | 1.56E-10 | 40 | 47 | BRT | T2 |
| Oryzomys chapmani | 0.043 | 0.00E+00 | 13 | 15 | GAM | T1 |
| Oryzomys chapmani | 0.0893 | 0.00E+00 | 40 | 47 | GAM | T2 |
| Oryzomys chapmani | 0.1793 | 0.00E+00 | 15 | 15 | GLM | T1 |
| Oryzomys chapmani | 0.3278 | 1.47E-14 | 40 | 47 | GLM | T2 |
| Oryzomys chapmani | 0.0276 | 0.00E+00 | 15 | 15 | Maxent | T1 |
| Oryzomys chapmani | 0.1595 | 0.00E+00 | 41 | 47 | Maxent | T2 |
| Oryzomys chapmani | 0.0623 | 0.00E+00 | 14 | 15 | RF | T1 |
| Oryzomys chapmani | 0.0586 | 0.00E+00 | 42 | 47 | RF | T2 |
| Oryzomys chapmani | 0.0958 | 0.00E+00 | 15 | 15 | SVM | T1 |
| Oryzomys chapmani | 0.1053 | 0.00E+00 | 41 | 47 | SVM | T2 |
| Oryzomys guerrerensis | 0.0086 | 2.77E-08 | 3 | 5 | Bioclim | T1 |
| Oryzomys guerrerensis | 0.0355 | 0.00E+00 | 6 | 6 | Bioclim | T2 |
| Oryzomys guerrerensis | 0.0821 | 0.00E+00 | 5 | 5 | BRT | T1 |
| Oryzomys guerrerensis | 0.0954 | 0.00E+00 | 6 | 6 | BRT | T2 |
| Oryzomys guerrerensis | 0.0638 | 1.06E-06 | 4 | 5 | GAM | T1 |
| Oryzomys guerrerensis | 0.0911 | 0.00E+00 | 6 | 6 | GAM | T2 |
| Oryzomys guerrerensis | 0.0786 | 3.01E-06 | 4 | 5 | GLM | T1 |
| Oryzomys guerrerensis | 0.7199 | 1.00E+00 | 0 | 6 | GLM | T2 |
| Oryzomys guerrerensis | 0.0048 | 2.67E-09 | 3 | 5 | Maxent | T1 |
| Oryzomys guerrerensis | 0.0804 | 2.71E-07 | 5 | 6 | Maxent | T2 |
| Oryzomys guerrerensis | 0.0399 | 1.23E-05 | 3 | 5 | RF | T1 |
| Oryzomys guerrerensis | 0.0588 | 0.00E+00 | 6 | 6 | RF | T2 |
| Oryzomys guerrerensis | 0.0295 | 0.00E+00 | 5 | 5 | SVM | T1 |
| Oryzomys guerrerensis | 0.0477 | 0.00E+00 | 6 | 6 | SVM | T2 |
| Oryzomys melanotis | 0.1683 | 6.70E-04 | 6 | 11 | Bioclim | T1 |
| Oryzomys melanotis | 0.0928 | 3.03E-04 | 4 | 8 | Bioclim | T2 |
| Oryzomys melanotis | 0.1541 | 0.00E+00 | 11 | 11 | BRT | T1 |
| Oryzomys melanotis | 0.3463 | 2.07E-04 | 7 | 8 | BRT | T2 |
| Oryzomys melanotis | 0.092 | 2.19E-08 | 8 | 11 | GAM | T1 |
| Oryzomys melanotis | 0.0733 | 3.82E-06 | 5 | 8 | GAM | T2 |
| Oryzomys melanotis | 0.1491 | 0.00E+00 | 11 | 11 | GLM | T1 |
| Oryzomys melanotis | 0.202 | 2.77E-06 | 7 | 8 | GLM | T2 |
| Oryzomys melanotis | 0.1018 | 1.22E-11 | 10 | 11 | Maxent | T1 |
| Oryzomys melanotis | 0.2029 | 9.31E-05 | 6 | 8 | Maxent | T2 |
| Oryzomys melanotis | 0.0498 | 4.66E-15 | 10 | 11 | RF | T1 |
| Oryzomys melanotis | 0.0731 | 8.31E-08 | 6 | 8 | RF | T2 |
| Oryzomys melanotis | 0.0888 | 0.00E+00 | 11 | 11 | SVM | T1 |
| Oryzomys melanotis | 0.1549 | 3.31E-07 | 7 | 8 | SVM | T2 |
| Osgoodomys banderanus | 0.3088 | 8.34E-08 | 21 | 27 | Bioclim | T1 |
| Osgoodomys banderanus | 0.2381 | 2.28E-13 | 27 | 33 | Bioclim | T2 |
| Osgoodomys banderanus | 0.4629 | 3.00E-08 | 25 | 27 | BRT | T1 |
| Osgoodomys banderanus | 0.3566 | 5.14E-07 | 25 | 33 | BRT | T2 |
| Osgoodomys banderanus | 0.2288 | 9.67E-06 | 16 | 27 | GAM | T1 |
| Osgoodomys banderanus | 0.2171 | 1.95E-14 | 27 | 33 | GAM | T2 |
| Osgoodomys banderanus | 0.3099 | 8.55E-09 | 22 | 27 | GLM | T1 |
| Osgoodomys banderanus | 0.4568 | 4.34E-04 | 24 | 33 | GLM | T2 |
| Osgoodomys banderanus | 0.2949 | 2.02E-10 | 23 | 27 | Maxent | T1 |
| Osgoodomys banderanus | 0.2835 | 2.72E-10 | 26 | 33 | Maxent | T2 |
| Osgoodomys banderanus | 0.1457 | 3.29E-13 | 20 | 27 | RF | T1 |
| Osgoodomys banderanus | 0.1246 | 4.11E-14 | 22 | 33 | RF | T2 |
| Osgoodomys banderanus | 0.3023 | 3.56E-10 | 23 | 27 | SVM | T1 |
| Osgoodomys banderanus | 0.1587 | 5.72E-13 | 23 | 33 | SVM | T2 |
| Pappogeomys bulleri | 0.1967 | 6.96E-11 | 15 | 17 | Bioclim | T1 |
| Pappogeomys bulleri | 0.0631 | 5.04E-02 | 1 | 6 | Bioclim | T2 |
| Pappogeomys bulleri | 0.2576 | 9.69E-11 | 16 | 17 | BRT | T1 |
| Pappogeomys bulleri | 0.1264 | 1.70E-01 | 1 | 6 | BRT | T2 |
| Pappogeomys bulleri | 0.1017 | 2.00E-15 | 15 | 17 | GAM | T1 |
| Pappogeomys bulleri | 0.0555 | 2.90E-01 | 0 | 6 | GAM | T2 |
| Pappogeomys bulleri | 0.246 | 2.34E-09 | 15 | 17 | GLM | T1 |
| Pappogeomys bulleri | 0.2802 | 5.59E-02 | 3 | 6 | GLM | T2 |
| Pappogeomys bulleri | 0.2426 | 0.00E+00 | 17 | 17 | Maxent | T1 |
| Pappogeomys bulleri | 0.2265 | 1.35E-04 | 5 | 6 | Maxent | T2 |
| Pappogeomys bulleri | 0.0591 | 3.66E-15 | 13 | 17 | RF | T1 |
| Pappogeomys bulleri | 0.0383 | 1.98E-02 | 1 | 6 | RF | T2 |
| Pappogeomys bulleri | 0.112 | 9.33E-15 | 15 | 17 | SVM | T1 |
| Pappogeomys bulleri | 0.0829 | 9.41E-03 | 2 | 6 | SVM | T2 |
| Peromyscus difficilis | 0.0806 | 0.00E+00 | 21 | 24 | Bioclim | T1 |
| Peromyscus difficilis | 0.149 | 0.00E+00 | 96 | 108 | Bioclim | T2 |
| Peromyscus difficilis | 0.1125 | 0.00E+00 | 22 | 24 | BRT | T1 |
| Peromyscus difficilis | 0.1791 | 0.00E+00 | 93 | 108 | BRT | T2 |
| Peromyscus difficilis | 0.035 | 0.00E+00 | 17 | 24 | GAM | T1 |
| Peromyscus difficilis | 0.088 | 0.00E+00 | 91 | 108 | GAM | T2 |
| Peromyscus difficilis | 0.0705 | 0.00E+00 | 21 | 24 | GLM | T1 |
| Peromyscus difficilis | 0.1258 | 0.00E+00 | 90 | 108 | GLM | T2 |
| Peromyscus difficilis | 0.0819 | 0.00E+00 | 22 | 24 | Maxent | T1 |
| Peromyscus difficilis | 0.1055 | 0.00E+00 | 96 | 108 | Maxent | T2 |
| Peromyscus difficilis | 0.0303 | 0.00E+00 | 16 | 24 | RF | T1 |
| Peromyscus difficilis | 0.055 | 0.00E+00 | 88 | 108 | RF | T2 |
| Peromyscus difficilis | 0.0641 | 0.00E+00 | 21 | 24 | SVM | T1 |
| Peromyscus difficilis | 0.1091 | 0.00E+00 | 92 | 108 | SVM | T2 |
| Peromyscus eva | 0.1339 | 8.02E-03 | 3 | 7 | Bioclim | T1 |
| Peromyscus eva | 0.3385 | 3.19E-08 | 21 | 25 | Bioclim | T2 |
| Peromyscus eva | 0.1255 | 4.91E-07 | 6 | 7 | BRT | T1 |
| Peromyscus eva | 0.2716 | 4.95E-09 | 20 | 25 | BRT | T2 |
| Peromyscus eva | 0.1529 | 1.18E-12 | 19 | 25 | GAM | T2 |
| Peromyscus eva | 0.0714 | 8.72E-07 | 5 | 7 | GLM | T1 |
| Peromyscus eva | 0.2387 | 2.24E-14 | 23 | 25 | GLM | T2 |
| Peromyscus eva | 0.1474 | 1.51E-06 | 6 | 7 | Maxent | T1 |
| Peromyscus eva | 0.1669 | 2.22E-16 | 22 | 25 | Maxent | T2 |
| Peromyscus eva | 0.0379 | 6.57E-05 | 3 | 7 | RF | T1 |
| Peromyscus eva | 0.1026 | 7.47E-12 | 16 | 25 | RF | T2 |
| Peromyscus eva | 0.0823 | 0.00E+00 | 7 | 7 | SVM | T1 |
| Peromyscus eva | 0.2246 | 5.33E-15 | 23 | 25 | SVM | T2 |
| Peromyscus furvus | 0.0373 | 1.43E-06 | 4 | 7 | Bioclim | T1 |
| Peromyscus furvus | 0.0558 | 0.00E+00 | 32 | 34 | Bioclim | T2 |
| Peromyscus furvus | 0.2646 | 9.09E-05 | 6 | 7 | BRT | T1 |
| Peromyscus furvus | 0.4947 | 1.44E-09 | 32 | 34 | BRT | T2 |
| Peromyscus furvus | 0.0582 | 2.59E-07 | 5 | 7 | GAM | T1 |
| Peromyscus furvus | 0.0245 | 0.00E+00 | 30 | 34 | GAM | T2 |
| Peromyscus furvus | 0.0698 | 8.04E-09 | 6 | 7 | GLM | T1 |
| Peromyscus furvus | 0.0811 | 0.00E+00 | 33 | 34 | GLM | T2 |
| Peromyscus furvus | 0.0531 | 1.49E-07 | 5 | 7 | Maxent | T1 |
| Peromyscus furvus | 0.03 | 0.00E+00 | 30 | 34 | Maxent | T2 |
| Peromyscus furvus | 0.0254 | 1.85E-09 | 5 | 7 | RF | T1 |
| Peromyscus furvus | 0.0285 | 0.00E+00 | 32 | 34 | RF | T2 |
| Peromyscus furvus | 0.0636 | 0.00E+00 | 7 | 7 | SVM | T1 |
| Peromyscus furvus | 0.0439 | 0.00E+00 | 27 | 34 | SVM | T2 |
| Peromyscus hylocetes | 0.0076 | 0.00E+00 | 4 | 4 | Bioclim | T1 |
| Peromyscus hylocetes | 0.018 | 5.12E-07 | 3 | 5 | Bioclim | T2 |
| Peromyscus hylocetes | 0.1124 | 0.00E+00 | 4 | 4 | BRT | T1 |
| Peromyscus hylocetes | 0.015 | 2.52E-07 | 3 | 5 | BRT | T2 |
| Peromyscus hylocetes | 0.0242 | 3.43E-07 | 3 | 4 | GAM | T1 |
| Peromyscus hylocetes | 0.0745 | 1.44E-04 | 3 | 5 | GAM | T2 |
| Peromyscus hylocetes | 0.0951 | 0.00E+00 | 4 | 4 | GLM | T1 |
| Peromyscus hylocetes | 0.0428 | 0.00E+00 | 5 | 5 | GLM | T2 |
| Peromyscus hylocetes | 0.062 | 0.00E+00 | 4 | 4 | Maxent | T1 |
| Peromyscus hylocetes | 0.0449 | 0.00E+00 | 5 | 5 | Maxent | T2 |
| Peromyscus hylocetes | 0.0128 | 0.00E+00 | 4 | 4 | RF | T1 |
| Peromyscus hylocetes | 0.0113 | 8.09E-08 | 3 | 5 | RF | T2 |
| Peromyscus hylocetes | 0.0389 | 0.00E+00 | 4 | 4 | SVM | T1 |
| Peromyscus hylocetes | 0.0342 | 0.00E+00 | 5 | 5 | SVM | T2 |
| Peromyscus levipes | 0.0272 | 4.29E-03 | 1 | 4 | Bioclim | T1 |
| Peromyscus levipes | 0.2268 | 0.00E+00 | 66 | 73 | Bioclim | T2 |
| Peromyscus levipes | 0.2874 | 0.00E+00 | 4 | 4 | BRT | T1 |
| Peromyscus levipes | 0.3066 | 0.00E+00 | 65 | 73 | BRT | T2 |
| Peromyscus levipes | 0.0663 | 1.11E-03 | 2 | 4 | GAM | T1 |
| Peromyscus levipes | 0.1199 | 0.00E+00 | 69 | 73 | GAM | T2 |
| Peromyscus levipes | 0.084 | 2.22E-03 | 2 | 4 | GLM | T1 |
| Peromyscus levipes | 0.1584 | 0.00E+00 | 69 | 73 | GLM | T2 |
| Peromyscus levipes | 0.1774 | 0.00E+00 | 4 | 4 | Maxent | T1 |
| Peromyscus levipes | 0.1464 | 0.00E+00 | 67 | 73 | Maxent | T2 |
| Peromyscus levipes | 0.1148 | 5.53E-03 | 2 | 4 | RF | T1 |
| Peromyscus levipes | 0.0713 | 0.00E+00 | 59 | 73 | RF | T2 |
| Peromyscus levipes | 0.1351 | 0.00E+00 | 4 | 4 | SVM | T1 |
| Peromyscus levipes | 0.1195 | 0.00E+00 | 63 | 73 | SVM | T2 |
| Peromyscus megalops | 0.0306 | 1.18E-10 | 7 | 11 | Bioclim | T1 |
| Peromyscus megalops | 0.0319 | 6.79E-14 | 9 | 12 | Bioclim | T2 |
| Peromyscus megalops | 0.0979 | 1.98E-05 | 6 | 11 | BRT | T1 |
| Peromyscus megalops | 0.1058 | 0.00E+00 | 12 | 12 | BRT | T2 |
| Peromyscus megalops | 0.0398 | 9.35E-10 | 7 | 11 | GAM | T1 |
| Peromyscus megalops | 0.0169 | 0.00E+00 | 10 | 12 | GAM | T2 |
| Peromyscus megalops | 0.0602 | 2.40E-08 | 7 | 11 | GLM | T1 |
| Peromyscus megalops | 0.0905 | 3.01E-13 | 11 | 12 | GLM | T2 |
| Peromyscus megalops | 0.0535 | 1.79E-10 | 8 | 11 | Maxent | T1 |
| Peromyscus megalops | 0.0521 | 8.75E-14 | 10 | 12 | Maxent | T2 |
| Peromyscus megalops | 0.029 | 2.41E-07 | 5 | 11 | RF | T1 |
| Peromyscus megalops | 0.0234 | 0.00E+00 | 11 | 12 | RF | T2 |
| Peromyscus megalops | 0.0548 | 2.23E-10 | 8 | 11 | SVM | T1 |
| Peromyscus megalops | 0.1258 | 1.57E-11 | 11 | 12 | SVM | T2 |
| Peromyscus melanocarpus | 0.004 | 0.00E+00 | 8 | 9 | Bioclim | T1 |
| Peromyscus melanocarpus | 0.0241 | 0.00E+00 | 16 | 20 | Bioclim | T2 |
| Peromyscus melanocarpus | 0.2201 | 5.88E-04 | 6 | 9 | BRT | T1 |
| Peromyscus melanocarpus | 0.466 | 5.57E-06 | 18 | 20 | BRT | T2 |
| Peromyscus melanocarpus | 0.0338 | 5.71E-14 | 8 | 9 | GAM | T1 |
| Peromyscus melanocarpus | 0.0214 | 0.00E+00 | 17 | 20 | GAM | T2 |
| Peromyscus melanocarpus | 0.0807 | 6.94E-07 | 6 | 9 | GLM | T1 |
| Peromyscus melanocarpus | 0.0466 | 0.00E+00 | 18 | 20 | GLM | T2 |
| Peromyscus melanocarpus | 0.0114 | 1.79E-10 | 5 | 9 | Maxent | T1 |
| Peromyscus melanocarpus | 0.0237 | 0.00E+00 | 16 | 20 | Maxent | T2 |
| Peromyscus melanocarpus | 0.006 | 3.68E-12 | 5 | 9 | RF | T1 |
| Peromyscus melanocarpus | 0.0134 | 0.00E+00 | 17 | 20 | RF | T2 |
| Peromyscus melanocarpus | 0.0182 | 2.28E-11 | 6 | 9 | SVM | T1 |
| Peromyscus melanocarpus | 0.0308 | 0.00E+00 | 18 | 20 | SVM | T2 |
| Peromyscus melanophrys | 0.1247 | 0.00E+00 | 69 | 84 | Bioclim | T1 |
| Peromyscus melanophrys | 0.1881 | 0.00E+00 | 60 | 67 | Bioclim | T2 |
| Peromyscus melanophrys | 0.1935 | 0.00E+00 | 67 | 84 | BRT | T1 |
| Peromyscus melanophrys | 0.2123 | 0.00E+00 | 56 | 67 | BRT | T2 |
| Peromyscus melanophrys | 0.1145 | 0.00E+00 | 70 | 84 | GAM | T1 |
| Peromyscus melanophrys | 0.1209 | 0.00E+00 | 58 | 67 | GAM | T2 |
| Peromyscus melanophrys | 0.1669 | 0.00E+00 | 71 | 84 | GLM | T1 |
| Peromyscus melanophrys | 0.1645 | 0.00E+00 | 59 | 67 | GLM | T2 |
| Peromyscus melanophrys | 0.1404 | 0.00E+00 | 74 | 84 | Maxent | T1 |
| Peromyscus melanophrys | 0.155 | 0.00E+00 | 59 | 67 | Maxent | T2 |
| Peromyscus melanophrys | 0.0657 | 0.00E+00 | 52 | 84 | RF | T1 |
| Peromyscus melanophrys | 0.0703 | 0.00E+00 | 45 | 67 | RF | T2 |
| Peromyscus melanophrys | 0.148 | 0.00E+00 | 73 | 84 | SVM | T1 |
| Peromyscus melanophrys | 0.1485 | 0.00E+00 | 56 | 67 | SVM | T2 |
| Peromyscus peRFulvus | 0.1334 | 1.33E-08 | 8 | 9 | Bioclim | T1 |
| Peromyscus peRFulvus | 0.2466 | 1.24E-03 | 5 | 7 | Bioclim | T2 |
| Peromyscus peRFulvus | 0.3853 | 0.00E+00 | 9 | 9 | BRT | T1 |
| Peromyscus peRFulvus | 0.4653 | 4.72E-03 | 6 | 7 | BRT | T2 |
| Peromyscus peRFulvus | 0.0603 | 0.00E+00 | 9 | 9 | GAM | T1 |
| Peromyscus peRFulvus | 0.0688 | 2.88E-05 | 4 | 7 | GAM | T2 |
| Peromyscus peRFulvus | 0.1574 | 0.00E+00 | 9 | 9 | GLM | T1 |
| Peromyscus peRFulvus | 0.3798 | 1.14E-03 | 6 | 7 | GLM | T2 |
| Peromyscus peRFulvus | 0.1254 | 0.00E+00 | 9 | 9 | Maxent | T1 |
| Peromyscus peRFulvus | 0.4643 | 0.00E+00 | 7 | 7 | Maxent | T2 |
| Peromyscus peRFulvus | 0.1116 | 0.00E+00 | 9 | 9 | RF | T1 |
| Peromyscus peRFulvus | 0.0991 | 9.38E-08 | 6 | 7 | RF | T2 |
| Peromyscus peRFulvus | 0.2022 | 0.00E+00 | 9 | 9 | SVM | T1 |
| Peromyscus peRFulvus | 0.2095 | 4.86E-04 | 5 | 7 | SVM | T2 |
| Peromyscus zarhynchus | 0.0499 | 1.82E-11 | 8 | 10 | Bioclim | T1 |
| Peromyscus zarhynchus | 0.0113 | 2.44E-15 | 7 | 9 | Bioclim | T2 |
| Peromyscus zarhynchus | 0.135 | 0.00E+00 | 10 | 10 | BRT | T1 |
| Peromyscus zarhynchus | 0.0708 | 8.76E-06 | 5 | 9 | BRT | T2 |
| Peromyscus zarhynchus | 0.0203 | 1.23E-12 | 7 | 10 | GAM | T1 |
| Peromyscus zarhynchus | 0.0263 | 2.02E-12 | 7 | 9 | GAM | T2 |
| Peromyscus zarhynchus | 0.062 | 0.00E+00 | 10 | 10 | GLM | T1 |
| Peromyscus zarhynchus | 0.03 | 2.76E-06 | 4 | 9 | GLM | T2 |
| Peromyscus zarhynchus | 0.0327 | 1.44E-15 | 9 | 10 | Maxent | T1 |
| Peromyscus zarhynchus | 0.0251 | 2.17E-10 | 6 | 9 | Maxent | T2 |
| Peromyscus zarhynchus | 0.071 | 0.00E+00 | 10 | 10 | RF | T1 |
| Peromyscus zarhynchus | 0.009 | 7.41E-09 | 4 | 9 | RF | T2 |
| Peromyscus zarhynchus | 0.0608 | 0.00E+00 | 10 | 10 | SVM | T1 |
| Peromyscus zarhynchus | 0.0291 | 4.73E-08 | 5 | 9 | SVM | T2 |
| Reithrodontomys chrysopsis | 0.0668 | 9.70E-14 | 11 | 13 | Bioclim | T1 |
| Reithrodontomys chrysopsis | 0.0256 | 4.65E-14 | 8 | 10 | Bioclim | T2 |
| Reithrodontomys chrysopsis | 0.0578 | 1.01E-10 | 9 | 13 | BRT | T1 |
| Reithrodontomys chrysopsis | 0.078 | 0.00E+00 | 10 | 10 | BRT | T2 |
| Reithrodontomys chrysopsis | 0.0342 | 2.05E-09 | 7 | 13 | GAM | T1 |
| Reithrodontomys chrysopsis | 0.0456 | 4.36E-08 | 6 | 10 | GAM | T2 |
| Reithrodontomys chrysopsis | 0.033 | 1.55E-09 | 7 | 13 | GLM | T1 |
| Reithrodontomys chrysopsis | 0.0731 | 4.37E-12 | 9 | 10 | GLM | T2 |
| Reithrodontomys chrysopsis | 0.0179 | 1.25E-13 | 8 | 13 | Maxent | T1 |
| Reithrodontomys chrysopsis | 0.0195 | 0.00E+00 | 9 | 10 | Maxent | T2 |
| Reithrodontomys chrysopsis | 0.013 | 7.11E-15 | 8 | 13 | RF | T1 |
| Reithrodontomys chrysopsis | 0.0291 | 0.00E+00 | 10 | 10 | RF | T2 |
| Reithrodontomys chrysopsis | 0.0207 | 3.77E-15 | 9 | 13 | SVM | T1 |
| Reithrodontomys chrysopsis | 0.0414 | 0.00E+00 | 10 | 10 | SVM | T2 |
| Sciurus alleni | 0.0288 | 1.81E-09 | 6 | 10 | Bioclim | T1 |
| Sciurus alleni | 0.0005 | 6.55E-10 | 2 | 4 | Bioclim | T2 |
| Sciurus alleni | 0.2416 | 3.22E-04 | 7 | 10 | BRT | T1 |
| Sciurus alleni | 0.077 | 3.52E-05 | 3 | 4 | BRT | T2 |
| Sciurus alleni | 0.0145 | 1.54E-11 | 6 | 10 | GAM | T1 |
| Sciurus alleni | 0.0494 | 5.96E-06 | 3 | 4 | GAM | T2 |
| Sciurus alleni | 0.3296 | 1.51E-05 | 9 | 10 | GLM | T1 |
| Sciurus alleni | 0.0626 | 1.54E-05 | 3 | 4 | GLM | T2 |
| Sciurus alleni | 0.0333 | 8.95E-06 | 4 | 10 | Maxent | T1 |
| Sciurus alleni | 0.0589 | 1.20E-05 | 3 | 4 | Maxent | T2 |
| Sciurus alleni | 0.0235 | 3.24E-08 | 5 | 10 | RF | T1 |
| Sciurus alleni | 0.0044 | 0.00E+00 | 4 | 4 | RF | T2 |
| Sciurus alleni | 0.0467 | 9.36E-10 | 7 | 10 | SVM | T1 |
| Sciurus alleni | 0.0027 | 0.00E+00 | 4 | 4 | SVM | T2 |
| Sciurus colliaei | 0.1715 | 3.94E-09 | 18 | 28 | Bioclim | T1 |
| Sciurus colliaei | 0.0095 | 3.99E-08 | 3 | 5 | Bioclim | T2 |
| Sciurus colliaei | 0.1297 | 0.00E+00 | 25 | 28 | BRT | T1 |
| Sciurus colliaei | 0.0344 | 6.83E-06 | 3 | 5 | BRT | T2 |
| Sciurus colliaei | 0.0429 | 1.62E-12 | 13 | 28 | GAM | T1 |
| Sciurus colliaei | 0.0533 | 3.85E-05 | 3 | 5 | GAM | T2 |
| Sciurus colliaei | 0.0773 | 0.00E+00 | 24 | 28 | GLM | T1 |
| Sciurus colliaei | 0.1097 | 1.59E-05 | 4 | 5 | GLM | T2 |
| Sciurus colliaei | 0.1001 | 0.00E+00 | 25 | 28 | Maxent | T1 |
| Sciurus colliaei | 0.0412 | 1.19E-07 | 4 | 5 | Maxent | T2 |
| Sciurus colliaei | 0.0469 | 0.00E+00 | 17 | 28 | RF | T1 |
| Sciurus colliaei | 0.0401 | 1.03E-07 | 4 | 5 | RF | T2 |
| Sciurus colliaei | 0.0855 | 0.00E+00 | 24 | 28 | SVM | T1 |
| Sciurus colliaei | 0.071 | 1.81E-06 | 4 | 5 | SVM | T2 |
| Sigmodon alleni | 0.1119 | 1.22E-04 | 5 | 9 | Bioclim | T1 |
| Sigmodon alleni | 0.0639 | 9.95E-04 | 2 | 4 | Bioclim | T2 |
| Sigmodon alleni | 0.0878 | 3.09E-10 | 8 | 9 | BRT | T1 |
| Sigmodon alleni | 0.1775 | 0.00E+00 | 4 | 4 | BRT | T2 |
| Sigmodon alleni | 0.038 | 2.30E-07 | 5 | 9 | GAM | T1 |
| Sigmodon alleni | 0.1292 | 0.00E+00 | 4 | 4 | GAM | T2 |
| Sigmodon alleni | 0.086 | 2.58E-10 | 8 | 9 | GLM | T1 |
| Sigmodon alleni | 0.1519 | 5.33E-04 | 3 | 4 | GLM | T2 |
| Sigmodon alleni | 0.0807 | 0.00E+00 | 9 | 9 | Maxent | T1 |
| Sigmodon alleni | 0.2074 | 0.00E+00 | 4 | 4 | Maxent | T2 |
| Sigmodon alleni | 0.0347 | 0.00E+00 | 9 | 9 | RF | T1 |
| Sigmodon alleni | 0.1953 | 0.00E+00 | 4 | 4 | RF | T2 |
| Sigmodon alleni | 0.1212 | 5.65E-09 | 8 | 9 | SVM | T1 |
| Sigmodon alleni | 0.0844 | 5.07E-05 | 3 | 4 | SVM | T2 |
| Sigmodon leucotis | 0.3092 | 7.78E-07 | 13 | 15 | Bioclim | T1 |
| Sigmodon leucotis | 0.3245 | 5.90E-03 | 5 | 7 | Bioclim | T2 |
| Sigmodon leucotis | 0.2527 | 4.96E-08 | 13 | 15 | BRT | T1 |
| Sigmodon leucotis | 0.2948 | 0.00E+00 | 7 | 7 | BRT | T2 |
| Sigmodon leucotis | 0.1119 | 1.27E-09 | 11 | 15 | GAM | T1 |
| Sigmodon leucotis | 0.1243 | 4.57E-07 | 6 | 7 | GAM | T2 |
| Sigmodon leucotis | 0.186 | 2.29E-08 | 12 | 15 | GLM | T1 |
| Sigmodon leucotis | 0.4874 | 0.00E+00 | 7 | 7 | GLM | T2 |
| Sigmodon leucotis | 0.1871 | 4.73E-07 | 11 | 15 | Maxent | T1 |
| Sigmodon leucotis | 0.416 | 0.00E+00 | 7 | 7 | Maxent | T2 |
| Sigmodon leucotis | 0.085 | 7.17E-07 | 8 | 15 | RF | T1 |
| Sigmodon leucotis | 0.2381 | 0.00E+00 | 7 | 7 | RF | T2 |
| Sigmodon leucotis | 0.2257 | 1.05E-08 | 13 | 15 | SVM | T1 |
| Sigmodon leucotis | 0.2978 | 0.00E+00 | 7 | 7 | SVM | T2 |
| Sigmodon mascotensis | 0.2534 | 9.26E-14 | 23 | 25 | Bioclim | T1 |
| Sigmodon mascotensis | 0.1705 | 0.00E+00 | 28 | 32 | Bioclim | T2 |
| Sigmodon mascotensis | 0.325 | 6.27E-13 | 24 | 25 | BRT | T1 |
| Sigmodon mascotensis | 0.2382 | 0.00E+00 | 29 | 32 | BRT | T2 |
| Sigmodon mascotensis | 0.1538 | 0.00E+00 | 24 | 25 | GAM | T1 |
| Sigmodon mascotensis | 0.1088 | 6.66E-16 | 22 | 32 | GAM | T2 |
| Sigmodon mascotensis | 0.376 | 2.40E-11 | 24 | 25 | GLM | T1 |
| Sigmodon mascotensis | 0.153 | 0.00E+00 | 29 | 32 | GLM | T2 |
| Sigmodon mascotensis | 0.1779 | 0.00E+00 | 24 | 25 | Maxent | T1 |
| Sigmodon mascotensis | 0.1506 | 0.00E+00 | 29 | 32 | Maxent | T2 |
| Sigmodon mascotensis | 0.0904 | 9.55E-13 | 16 | 25 | RF | T1 |
| Sigmodon mascotensis | 0.0592 | 0.00E+00 | 19 | 32 | RF | T2 |
| Sigmodon mascotensis | 0.2656 | 2.05E-10 | 21 | 25 | SVM | T1 |
| Sigmodon mascotensis | 0.1684 | 0.00E+00 | 27 | 32 | SVM | T2 |
| Thomomys atrovarius | 0.1224 | 2.76E-06 | 8 | 13 | Bioclim | T1 |
| Thomomys atrovarius | 0.0311 | 5.55E-03 | 1 | 4 | Bioclim | T2 |
| Thomomys atrovarius | 0.1923 | 0.00E+00 | 13 | 13 | BRT | T1 |
| Thomomys atrovarius | 0.1208 | 2.13E-04 | 3 | 4 | BRT | T2 |
| Thomomys atrovarius | 0.0478 | 1.55E-11 | 9 | 13 | GAM | T1 |
| Thomomys atrovarius | 0.0597 | 8.14E-04 | 2 | 4 | GAM | T2 |
| Thomomys atrovarius | 0.0895 | 0.00E+00 | 13 | 13 | GLM | T1 |
| Thomomys atrovarius | 0.0785 | 1.82E-03 | 2 | 4 | GLM | T2 |
| Thomomys atrovarius | 0.1086 | 0.00E+00 | 13 | 13 | Maxent | T1 |
| Thomomys atrovarius | 0.1995 | 0.00E+00 | 4 | 4 | Maxent | T2 |
| Thomomys atrovarius | 0.0688 | 7.77E-16 | 12 | 13 | RF | T1 |
| Thomomys atrovarius | 0.0395 | 2.40E-04 | 2 | 4 | RF | T2 |
| Thomomys atrovarius | 0.056 | 0.00E+00 | 12 | 13 | SVM | T1 |
| Thomomys atrovarius | 0.1218 | 2.20E-04 | 3 | 4 | SVM | T2 |

Table S5. Mean value and standard deviation of performance metrics for the niche models transferred to past (hindcast) and future (forecast) climatic scenarios for seven modeling algorithms. N = number of species; OI =  Overlap Index; TSS = True Skill Statistics; FNR = False Negative Rate; False Positive Rate. Modeling algorithms: Bioclim, Generalized Additive Models (GAM), Generalized Linear Models (GLM), Boosted Regression Trees (BRT), Maxent, Random Forest (RF), and Support Vector Machine (SVM).

| Direction | Model | N | OI | TSS | FNR | FPR |
| --- | --- | --- | --- | --- | --- | --- |
| Forecast | Bioclim | 38 | 0.49 ± 0.25 | 0.47 ± 0.25 | 0.51 ± 0.25 | 0.02 ± 0.02 |
| BRT | 39 | 0.93 ± 0.06 | 0.85 ± 0.08 | 0.07 ± 0.06 | 0.08 ± 0.07 |
| GAM | 41 | 0.84 ± 0.01 | 0.55 ± 0.4 | 0.16 ± 0.01 | 0.29 ± 0.4 |
| GLM | 36 | 0.77 ± 0.17 | 0.73 ± 0.15 | 0.23 ± 0.17 | 0.05 ± 0.03 |
| Maxent | 38 | 0.78 ± 0.15 | 0.75 ± 0.14 | 0.22 ± 0.15 | 0.03 ± 0.03 |
| RF | 42 | 0.86 ± 0.12 | 0.82 ± 0.1 | 0.14 ± 0.12 | 0.04 ± 0.03 |
| SVM | 42 | 0.8 ± 0.16 | 0.77 ± 0.14 | 0.2 ± 0.16 | 0.03 ± 0.03 |
| Hindcast | Bioclim | 40 | 0.65 ± 0.18 | 0.61 ± 0.16 | 0.35 ± 0.18 | 0.04 ± 0.03 |
| BRT | 39 | 0.8 ± 0.19 | 0.73 ± 0.16 | 0.2 ± 0.19 | 0.07 ± 0.09 |
| GAM | 41 | 0.86 ± 0.09 | 0.81 ± 0.08 | 0.14 ± 0.09 | 0.06 ± 0.03 |
| GLM | 35 | 0.87 ± 0.07 | 0.77 ± 0.08 | 0.13 ± 0.07 | 0.1 ± 0.07 |
| Maxent | 38 | 0.86 ± 0.13 | 0.82 ± 0.12 | 0.14 ± 0.13 | 0.04 ± 0.04 |
| RF | 42 | 0.89 ± 0.07 | 0.81 ± 0.08 | 0.11 ± 0.07 | 0.09 ± 0.06 |
| SVM | 42 | 0.82 ± 0.13 | 0.75 ± 0.11 | 0.18 ± 0.13 | 0.08 ± 0.1 |

B

Figure S1. Mobility-Oriented Parity (MOP) analysis (Owens et al., 2013) between the mid-20th century (Time 1 [T1]: 1950-1979) and the late 20th/early 21st centuries (Time 2 [T2]: 1980-2009) climatic datasets (Cuervo-Robayo et al., 2020). Areas in red represent non-analog climates where strict extrapolation is required. (A) T1 vs T2. (B) T2 vs T1. MOP analysis was conducted using the smop package (Osorio-Olvera & Contreras-Díaz, 2024) in R, and the maps were created with QGIS 3.36 (QGIS.org, 2023).

A

References

Cuervo-Robayo, Á., Ureta, C., Gómez-Albores, M.A., Meneses Mosquera, A.K., Téllez Valdés, O., Martínez-Meyer, E. 2020. One hundred years of climate change in Mexico. PLoS ONE 15:1–19. DOI: 10.1074/jbc.RA118.003614.

Osorio-Olvera, L & Contreras-Díaz, R.G. 2024. smop. Simple but Fast MOP Analysis ver. 0.0.2. https://luismurao.github.io/smop/.

Owens, H. L., Campbell, L. P., Dornak, L. L., Saupe, E. E., Barve, N., Soberón, J.,Ingenloff K., Lira-Noriega A., Hensz C.M., Myers C.E.,Peterson AT. 2013. Constraints on interpretation of ecological niche models by limited environmental ranges on calibration areas. Ecological modelling, 263, 10-18.[https://doi.org/10.1016/j.ecolmodel.2013.04.011](https://doi.org/10.1016/j.ecolmodel.2013.04.011).

QGIS.org, 2023. QGIS 3.36 Geographic Information System. QGIS Association. http://www.qgis.org.
